# Inter-subject correlations of EEG reflect subjective arousal and acoustic features of music

**DOI:** 10.3389/fnhum.2023.1225377

**Published:** 2023-08-21

**Authors:** Fuyu Ueno, Sotaro Shimada

**Affiliations:** ^1^Department of Electronics and Bioinformatics, School of Science and Technology, Meiji University, Kawasaki, Japan; ^2^Japan Society for the Promotion of Science, Tokyo, Japan

**Keywords:** inter-subject correlation, EEG, music listening, emotion, clustering

## Abstract

**Background:**

Research on music-induced emotion and brain activity is constantly expanding. Although studies using inter-subject correlation (ISC), a collectively shared brain activity analysis method, have been conducted, whether ISC during music listening represents the music preferences of a large population remains uncertain; additionally, it remains unclear which factors influence ISC during music listening. Therefore, here, we aimed to investigate whether the ISCs of electroencephalography (EEG) during music listening represent a preference for music reflecting engagement or interest of a large population in music.

**Methods:**

First, we selected 21 pieces of music from the Billboard Japan Hot 100 chart of 2017, which served as an indicator of preference reflecting the engagement and interest of a large population. To ensure even representation, we chose one piece for every fifth song on the chart, spanning from highly popular music to less popular ones. Next, we recorded EEG signals while the subjects listened to the selected music, and they were asked to evaluate four aspects (preference, enjoyment, frequency of listening, and arousal) for each song. Subsequently, we conducted ISC analysis by utilizing the first three principal components of EEG, which were highly correlated across subjects and extracted through correlated component analysis (CorrCA). We then explored whether music with high preferences that reflected the engagement and interest of large population had high ISC values. Additionally, we employed cluster analysis on all 21 pieces of music, utilizing the first three principal components of EEG, to investigate the impact of emotions and musical characteristics on EEG ISC during music listening.

**Results:**

A significant distinction was noted between the mean ISC values of the 10 higher-ranked pieces of music compared to the 10 lower-ranked pieces of music [*t*(542) = −1.97, *p* = 0.0025]. This finding suggests that ISC values may correspond preferences reflecting engagement or interest of a large population. Furthermore, we found that significant variations were observed in the first three principal component values among the three clusters identified through cluster analysis, along with significant differences in arousal levels. Moreover, the characteristics of the music (tonality and tempo) differed among the three clusters. This indicates that the principal components, which exhibit high correlation among subjects and were employed in calculating ISC values, represent both subjects’ arousal levels and specific characteristics of the music.

**Conclusion:**

Subjects’ arousal values during music listening and music characteristics (tonality and tempo) affect ISC values, which represent the interest of a large population in music.

## 1. Introduction

The interpretation and appreciation of music requires extensive bilateral control of attention, memory, emotion, reward, motor skills, and auditory, syntactic, and semantic processing, organized by a complex brain network centered in the temporal lobe but spanning multiple cortical and subcortical regions (Sihvonen and Särkämö, 2022). In the brain, the interpretation of higher-order musical integrative features such as chords and harmonies occurs in multiple frontal and parietal lobe regions, including the inferior frontal gyrus (IFG), medial prefrontal cortex, inferior parietal lobule, and premotor areas (Janata et al., 2002; Schulze et al., 2011; Foster et al., 2013; Royal et al., 2016). Moreover, the perception of rhythm involves a motor network consisting of the cerebellum, basal ganglia, and primary motor cortex (Grahn and Brett, 2007; Chen et al., 2008). In addition, the thalamus projects sound information to limbic areas such as the amygdala and orbitofrontal cortex, allowing for rapid analysis of emotional acoustic cues in music (LeDoux, 2000). These limbic structures have been found to engage in repetitive exchanges with the auditory cortex, facilitating the cortical elaboration of auditory signals (Frühholz et al., 2016) and generating music’s hedonic and motivational value (Salimpoor et al., 2013).

Recent advances in statistical methods and meta-analysis have allowed the incorporation of big data into human imaging (Pando-Naude et al., 2021). Using techniques of the activation likelihood estimation (ALE; Laird et al., 2010) methodology, a coordinate-based algorithm for meta-analysis of neuroimaging studies, Pando-Naude et al. (2021) conducted a coordinate-based meta-analysis of a wide range of functional magnetic resonance imaging (fMRI) studies. The results revealed that music perception involves the right superior temporal gyrus (STG), left superior frontal gyrus, left medial frontal gyrus, right lentiform nucleus (putamen), left lentiform nucleus (putamen), caudate, left cerebellum, left insula, and right frontal gyrus (Pando-Naude et al., 2021). It was inferred that cortical and subcortical areas that process musical information both “bottom-up” (sensory processing is influenced by the narrative nature of the music) and “top-down” (individual preferences may alter an individual’s level of attention and engagement) are hierarchically organized (Pando-Naude et al., 2021). In addition, music imagery was found to involve the left medial frontal gyrus, left superior parietal lobule, left thalamus, and left frontal gyrus (Pando-Naude et al., 2021). Music imagery recruits motor areas involved in generating movement, such as the premotor and supplementary motor areas, areas of the basal ganglia also involved in facilitating movement, and parietal areas involved in perceptual-motor coordination and theory-of-mind (Pando-Naude et al., 2021).

With the mechanisms of how the brain processes music being clarified, the study of music-induced emotion and brain activity is constantly increasing (Koelsch, 2020), and new findings are being obtained using a variety of analysis methods. Using a data-driven approach with group independent component analysis (ICA), sliding time-window correlation, and k-means clustering, Liu et al. (2021) analyzed the spatial connectivity and temporal dynamic functional network connectivity (dFNC) of emotions evoked by a dynamically changing tempo. The results showed that music with decreasing tempo enhanced FNC in the default mode network, sensorimotor network, and frontoparietal network, strengthening neural networks in emotional processing to keep listeners in a stable, pleasant state (Liu et al., 2021). On the contrary, music with an increasing tempo was found to be less potent in evoking multiple neural networks and made listeners’ emotional processing unstable (Liu et al., 2021). In addition, Daly et al. (2019) simultaneously recorded electroencephalography (EEG) and fMRI during music listening and investigated how EEG-based emotional responses to music reflect changes in activity in the subcortical emotional response network measured by fMRI by measuring EEG asymmetry in the prefrontal cortex. The results indicated that EEG asymmetry in the prefrontal cortex is significantly related to the activity of subcortical emotional response networks, including the amygdala, posterior temporal cortex, and cerebellum (Daly et al., 2019).

Furthermore, the fact that music induces pleasant sensations has recently attracted attention in the field of neuroscience (Mas-Herrero et al., 2021a). A meta-analysis study and a study combining transcranial magnetic stimulation with fMRI suggested that music-induced pleasure involves both higher-order cortical regions, such as the right STG and right IFG, which are concerned with auditory perception and predictive encoding, and reward-related regions such as the striatum (Mas-Herrero et al., 2021a,b). Furthermore, Ara and Marco-Pallarés (2020) investigated the EEG synchronization underlying the pleasant sensations associated with listening to music using a multilevel Bayesian approach. As a result, phase synchronization between the right temporal and frontal theta bands was shown to play an important role in the pleasant sensations associated with listening to music (Ara and Marco-Pallarés, 2020).

However, existing studies on brain activity during music-induced emotional and pleasurable arousal have not reached a unified view, as the brain areas and functional connectivity that have been suggested to be associated with musical emotions and pleasurable feelings are not consistent. For example, Mas-Herrero et al. (2021b) showed that the bilateral insular cortex (INS), bilateral STG, right IFG, bilateral ventral striatum, anterior prefrontal cortex, and ventromedial prefrontal cortex (vmPFC) were brain regions involved when listeners reported a pleasurable music experience. However, Salimpoor et al. (2013) found that functional connectivity of the nucleus accumbens and STG increased very robustly when subjects were listening to music that they rated as having the highest reward value. Furthermore, hemodynamic increases in the vmPFC, orbitofrontal cortex (OFC), and amygdala did not predict changes in the reward value of music. In addition, previous studies on music-induced emotions have revealed correlations between the intensity of chills and regional cerebral blood flow in the INS (Blood and Zatorre, 2001), and blood oxygenation in the INS increases during music listening, which causes high emotional valence and high arousal (Trost et al., 2012). However, a coordinate-based ALE meta-analysis of music-induced emotions did not identify clusters in the INS (Koelsch, 2020).

Therefore, we focused on inter-subject correlation (ISC) analysis (Hasson et al., 2004), which has recently facilitated the elucidation of the mechanisms by which the brain processes natural stimuli. ISC analysis is a method of collectively analyzing shared brain activity, which can be used to quantify the degree to which a subject’s brain activity is similar to that of other subjects (Hasson et al., 2004; Simony et al., 2016). Previous studies on ISCs to natural stimuli used audiovisual stimuli such as film (Hasson et al., 2008; Kauppi et al., 2010; Dmochowski et al., 2012; Tu et al., 2019; Bolton et al., 2020; Gruskin et al., 2020; Patel et al., 2021; Ou et al., 2022), television/video (Cantlon and Li, 2013; Schmälzle et al., 2013; Chen and Farivar, 2020; Kotila et al., 2021), stories (Wilson et al., 2008; Finn et al., 2018; Lerner et al., 2018; Cohen et al., 2022), speech (Schmälzle et al., 2015), and audio (Kandeepan et al., 2020; Thiede et al., 2020).

For example, Hasson et al. (2004) used fMRI to measure brain activity while participants viewed the movie “The Good, the Bad and The Ugly” and conducted an ISC analysis. Specifically, they computed pairwise correlations of the fMRI signals of different subjects and averaged the results at the group level (Hasson et al., 2004; Trost et al., 2015). In doing so, they identified brain regions that showed similar activation time courses across subjects without *a priori* defining events (Hasson et al., 2004; Trost et al., 2015). They found similar responses between subjects in the visual and auditory cortex and frontal and parietal lobes (Hasson et al., 2004). This suggests that the film stimulated each viewer’s senses and perceptions and the same systems were used to understand the story and emotional response to events (Schmälzle and Grall, 2020). Following this study, Dmochowski et al. (2014) suggested that there are correlations between the ISC and the frequency of an episode of TV being tweeted about (the rate of tweets associated with each scene or episode), and between the ISC and Nielsen ratings (time series in minutes) when watching popular TV programs. Furthermore, they also conjectured that the strength of ISCs of EEGs obtained from a relatively small number of subjects (about a dozen) while watching 20 different commercials can predict the preferences of large audiences across the United States for commercials (Dmochowski et al., 2014). Thus, ISCs of brain activity in a small group can be used to predict public behavioral responses (Dmochowski et al., 2014).

Inter-subject correlation analysis is also considered to be a useful approach in the study of music listening. This is because emotional experiences shared by different people at the same moment toward a piece of music may lead to increased synchrony of brain activation patterns among listeners. Moreover, when ISC occurs at a particular moment in a piece of music, it may reflect the music’s acoustic characteristics or the music’s generation of subjective emotions (Trost et al., 2015). Therefore, ISC analysis is considered a powerful data-driven methodology that may provide key information about music processing and music-induced emotions by extracting meaningful information from ongoing brain activity under natural conditions (Trost et al., 2015).

Previous studies using ISC analysis have evaluated various aspects of music perception. In a study on acoustic feature processing, Alluri et al. (2012) investigated the neural correlates of timbre, tonal, and rhythmic feature processing to naturalistic music stimuli and identified large-scale cognitive, motor, and limbic brain circuits dedicated to acoustic feature processing. Regarding the neural processing of music, Abrams et al. (2013) suggested that naturalistic music elicits synchronized patterns of neural activity across individuals in auditory, motor, and frontal regions of the brain associated with higher cognitive functions and that the structure of the musical sequence alters the synchronization of this entire network. Farbood et al. (2015) showed that the processing time scale of music gradually lengthens toward higher brain regions. Regarding the relationship between ISCs and behavioral ratings, Kaneshiro et al. (2020) found that, while the original stimuli of popular Bollywood movie music were subjectively rated as most pleasant, the highest ISC values were found in the time-scrambled (measure shuffling) stimulus. Furthermore, Dauer et al. (2021) found that a popular music-style remix of Steve Reich’s “Piano Phase,” a minimal music piece, caused higher ISCs of EEGs and higher ISCs of continuous behavior than the original piece; namely, the abrupt change stimulus [no phasing section (phase shift)] and segment shuffle stimuli (segments switched by 5 s each). Finally, with regard to musical emotional processing, Trost et al. (2015) observed significant synchronization between listeners in a distributed brain network that included not only the auditory cortex but also regions associated with visual, motor, attentional, and emotive processes, and brain activation during the synchronization period was associated with various acoustic features in the music. Sachs et al. (2020) showed that enjoyment of sad music predicted inter-subject synchrony in the auditory cortex, basal ganglia regions, OFC, and posterior cingulate.

Thus, ISC analysis is being used to decipher brain states during music listening. Schubert et al. (2013) suggest that the ISC reflects that listeners are drawn to music and interested in what happens next, and Dmochowski et al. (2014) suggest that the ISC represents preferences of large audiences reflecting engagement or interest in video advertising. However, it is unclear whether ISC during music listening, as opposed to video content, also represent preferences reflecting engagement or interest of a large population. In addition, as mentioned earlier, while a growing number of studies on ISC analysis of neural responses during music listening, the stimulus attributes that contribute to neural correlates are still poorly understood (Kaneshiro et al., 2021). In other words, it is not clear what factors influence ISC during music listening.

Here, we aimed to investigate whether the ISCs of EEGs during music listening represent a preference for music reflecting engagement or interest of a large population in music. Using a commercial music chart that indicates the music preferences of a large population (reflecting engagement and interest), we selected pieces of music and conducted an ISC analysis of EEGs measured during music listening. In this study, we found the linear component of the data that maximizes the cross-correlation of EEG signals between different subjects (CorrCA; Dmochowski et al., 2012). The ISC values were then statistically calculated using a general linear model (GLM) for the degree of fitting between the components. Then, we investigated whether the ISCs of EEG during music listening represents a preference for music reflecting engagement or interest of a large population. Furthermore, by conducting a cluster analysis of the EEG correlation components between subjects as features, we investigated which emotional and musical characteristics influenced the ISCs of EEGs while listening to music.

## 2. Materials and methods

### 2.1. Participants

Seventeen healthy adults participated in the experiment [7 females, aged 21.4 ± 0.69 years, mean ± standard deviation (SD)]. Of the 17 participants, 13 were students at Meiji University, and four were young adults living in the university’s neighborhood. To ensure that the results of the experiment were unbiased to the musical training or ability of the participants, they completed a questionnaire about their background in music listening and performance. Fifteen of the 17 participants indicated that they usually listen to music (1.768 ± 1.207 h/day). Although none of the participants were professional musicians, six participants had musical experience, with a mean of 7.167 ± 5.336 years. All participants had normal hearing and provided written informed consent to participate in this study. The study protocol was approved by the Ethics Committee of the School of Science and Technology, Meiji University. This study was conducted according to the principles and guidelines of the Declaration of Helsinki.

### 2.2. EEG recordings

The BCI Research System, an EEG measurement system manufactured by g.tec, was used for EEG measurements. A bioamplifier for EEG measurement (g.USBamp; g.tec, Schiedlberg, Austria) was used for EEG, and sintered Ag/AgCl electrodes from the same measurement system were used for the electrodes. The electrodes were an active scalp electrode (g.LADYbird, g.tec) and a reference electrode (g.GAMMAearclip Ag/AgCl, g.tec). The scalp and ground electrodes were flat electrodes, and the reference electrode was an ear-clip type. A gamma box for direct current (g.GAMMAbox for 16 channels DC, g.tec) was used as the connection device between the bioamplifier and the electrodes, and a connector cable (g.USBampGAMMAconnector, g.tec) was used for the connection. Electrode caps (g.EEGcap, g.tec) were used to attach the electrodes in the positions specified by the international 10–20 system. The electrode cap was size M (for a head circumference of 540–580 mm). A special highly conductive and highly adhesive gel (g.GAMMAgel, g.tec) was inserted between the electrode and the skin to keep the impedance between the electrode and the skin below 10 kΩ. In addition, disposable electrocardiography (ECG) electrodes (SENSTEC, Japan) were attached to clip-on electrodes (g.GAMMAclip, g.tec), and electro-oculography (EOG) was measured simultaneously with EEG measurements from the same biological amplifier. A 0.5–100 Hz bandpass filter was applied to the EEG and EOG, and the sampling frequency was recorded at 512 Hz.

Two PCs were used in the experiment: a measurement PC to measure EEG data and a control PC to run the experimental program. The bioamplifiers were controlled and measured using numerical analysis software (MATLAB; MathWorks, Natick, MA) and Simulink on the measurement PC. Experimental programs were created and executed on the control PC using psychological experiment software (E-prime 3.0; Psychology Software Tools, Sharpsburg, PA). The software was also used to present the musical stimuli and to acquire the trigger signal.

In EEG measurements, electrodes were mounted in 30 positions according to the international 10–20 system (Fp1, Fpz, Fp2, F7, F3, Fz, F4, F8, FC3, T7, C5, C3, Cz, C4, C6, T8, TP7, CP5, CP3, CP4, CP6, TP8, P7, P5, P3, Pz, P4, P6, P8, O1, and O2). First, Cz was determined from the midpoint of the line connecting the nasal root and occipital tubercle and the midpoint connecting the left and right anterior auricular points. Then, an electrode cap was placed over the entire head with Cz as the reference. The ground electrode was located at AFz, the reference was mounted on the right earlobe, and the montage was recorded using the reference electrode derivation method. Vertical EOGs were recorded from above and below the right eye.

### 2.3. Music stimuli

Experimental stimuli were selected from the Billboard Japan Hot 100 chart of 2017. The Billboard Japan Hot 100 is a composite chart that references not only indicators such as the number of CD sales and music downloads, but also the number of video views on YouTube and other media, and the number of tweets about songs and artists’ names. This chart was used in this study because Dmochowski et al. (2014) utilized online networks such as Facebook and Twitter to assess public behavioral responses (including preferences). To evenly represent preferences reflecting engagement or interest of a large population, every fifth song from the chart was selected, for a total of 21 songs. The list of all 21 pieces of music is provided in Supplementary Table 1. Each piece of music was imported from a music CD as an mp3 file into audio editing software (Audacity 2.3.2^1^) and edited to 62 s (1 s at the beginning of the music: fade in, 1 s at the end of the music: fade out). The chorus was always included as it is the part that impresses people as the main motif in the song, and the verse, bridge, and other parts that are connected before and after the chorus were also included. No other techniques of loudness normalization or other editing were used, and the mean loudness of all songs was −16.662 ± 1.376 dBFS.

### 2.4. Procedure

E-prime 3.0 (Psychology Software Tools) was used to control the experimental procedure. In addition, a 27-inch LCD monitor and mouse were used to present the fixation cross and collect input for subjective evaluation. Participants were seated in front of the monitor, and their EEGs were measured while listening to the presented music. The subjects were asked to use the earphones they normally use, but those who did not have any used earphones from the laboratory (Hi-Res in Colors; ELECOM, Osaka, Japan).

During each trial, participants were instructed to gaze on a fixation cross. After 5 s of silence, the music stimulus was presented for 62 s followed by silence for 5 s. Participants indicated their degree of preference for the music by sliding the mouse along an 11-point scale from −5 to 5. As soon as the participant finished answering, the participant clicked the button marked “Next” to start the next trial. Participants were allowed to take a short break as needed between trials. The 21 selected songs were presented randomly to the participants. The EEG measurement was finished when participants had completed all trials. Then, the participants listened to the 62-s clip of each song again. This time, the participants indicated their degree of preference, enjoyment, frequency of listening, and arousal on an 11-point scale from −5 to 5 using a mouse. Hence, only the preference evaluation was conducted twice because we used this to check for any changes in the evaluation between EEG measurement and questionnaire answering. The entire experiment took approximately 1 h, including EEG measurements during music listening and questionnaire answering.

### 2.5. Data analysis

Electroencephalography data were preprocessed using MATLAB (MathWorks) and EEGLAB 14.1.1b (Swartz Center for Computational Neuroscience, San Diego, CA). First, raw data were subjected to a 1–60 Hz bandpass filter and a 50-Hz notch filter. Next, the data were subjected to ICA and elimination of ocular artifacts. After that, we performed correlated component analysis (CorrCA) (Dmochowski et al., 2012; Jäncke and Alahmadi, 2016), ISC analysis (Hasson et al., 2004; Simony et al., 2016), and clustering of brain activity components using preprocessed data.

First, we performed CorrCA to extract only wave patterns expressing specific brain activity with high correlation between subjects as components. CorrCA is a dimensional compression method for integrating signals by extracting only those signals common to multiple sources of information (Dmochowski et al., 2012). While principal component analysis, a well-known representative method of dimensional compression, takes the maximum variance among a given data set, CorrCA takes as its component the maximum correlation among data sets. This concept of maximizing correlations is identical to canonical correlation analysis (Hotelling, 1936), the only difference being that the data sets are in the same space and share the same projection vector. CorrCA calculates weights w, maximizing Pearson’s correlation coefficient between components x1 and x2. In the next ISC analysis, the ISC value is calculated for the component y by multiplying the EEG data X and the weight w obtained from CorrCA. We calculated the common weights that are unified for all subjects and all music up to 30 components using CorrCA. Specifically, we first concatenated the data of all music for each subject. At that time, the baseline was corrected to be equal to the values at the end and the beginning of the data to smooth the connection. Subsequently, we performed CorrCA using the concatenated data of all subjects, and weights w were calculated. We employed the CorrCA approach of Dmochowski et al. (2012), the mathematical details of which are described below.

The EEG data X is defined as *X*_1_ ∈ *R*^*D*×*T*^ and *X*_2_ ∈ *R*^*D*×*T*^ with the number of channels *D* and the number of time samples *T*. The transposed EEG data multiplied by the weight *w* are $y_{1}=X_{1}^{T}\,w$ and $y_{2}=X_{2}^{T}\,w$, where the weight vector is *w*_1_ ∈ ℝ^*D*^. We found the weight vector $\hat{w}$ so that *y_1_* and *y_2_* were maximally correlated:


(1) $$\hat{\text{w}}=\text{arg}\,\max_{w}\frac{{\text{y}}_{1}^{T}\,{\text{y}}_{2}}{||{\text{y}}_{1}||\,||{\text{y}}_{2}||}=\text{arg}\,\max_{w}\frac{{\text{w}}^{T}\,{\text{R}}_{12}\,\text{w}}{\sqrt{{\text{w}}^{T}\,{\text{R}}_{11}\,\text{w}}\,\sqrt{{\text{w}}^{T}\,{\text{R}}_{22}\,\text{w}}},$$

where the covariance matrices are denoted by $R_{11}=\frac{1}{T}\,X_{1}\,X_{1}^{T}$, $R_{22}=\frac{1}{T}\,X_{2}\,X_{2}^{T}$ and $R_{12}=\frac{1}{T}\,X_{1}\,X_{2}^{T}$. The differentiating equation (1) with respect to *w* and setting it to zero led to the following equation:


(2) $$\frac{\sigma_{11}\,\sigma_{22}}{\sigma_{12}}\,{\text{R}}_{12}\,\text{w}=\left(\sigma_{22}\,{\text{R}}_{11}+\sigma_{11}\,{\text{R}}_{22}\right)\,\text{w},$$

where σ_11_ = *w^T^R*_11_*w*, σ_22_ = *w^T^R*_22_*w* and σ_12_ = *w^T^R*_12_*w* are scalar power terms to bring the two data sets onto the same scale. Here we assume that the two data sets have similar power levels, σ_11_≈σ_22_. Furthermore, symmetrizing the mutual covariance matrix *R*_*12*_ leads to the following eigenvalue equation:


(3) $${\left({\text{R}}_{11}+{\text{R}}_{22}\right)}^{-1}\,\left({\text{R}}_{12}+{\text{R}}_{21}\right)\,\text{w}=\lambda \,\text{w},$$

where λ=σ_22_/σ_11_. The eigenvector obtained by solving the eigenvalue equation is $\hat{w}$. The eigenvector for the largest eigenvalue (i.e., highest correlation) is the first principal component, the eigenvector for the second largest eigenvalue is the second principal component, and so on.

We obtained spatial filters corresponding to brain regions using the forward model (Parra et al., 2005) to examine the brain activity characteristics for each principal component. Then, the absolute values were taken and normalized so that the maximum value was 1 for each principal component, and the brain area for each principal component was described as a color map. The forward model is presented by the following equation A:


(4) $$\text{A}=\text{RW}\,{\left({\text{W}}^{T}\,\text{RW}\right)}^{-1}$$

Second, we performed ISC analysis to quantitatively acquire a similar degree of brain activity between subjects. We used a GLM to obtain the ISC value (Hasson et al., 2004; Simony et al., 2016). GLM is an analysis method that statistically examines how well the observed signal data can be fitted with a design matrix model (Friston et al., 1994). We fitted component *y_1_* that multiplied the EEG data of one subject *X_1_* by the weight *w* as the design matrix to component *y_2_* that multiplied the EEG data of one subject *X_2_* by the weight *w*. When the component data of the modeled subject was *y_1_*, GLM was used in the following equation and described the same component data *y_2_* of other subjects:


(5) $${\text{y}}_{2}={\text{y}}_{1}\,\beta +\text{e},$$

where β is the model parameter, and *e* is the error term. How well the observed EEG data (*y_2_*) can be fitted by the design matrix (*y_1_*), that is, whether the data (*y_2_*) can be explained by the model (*y_1_*) with sufficient accuracy, depends on whether this error term is sufficiently small. The least squares estimator *b* of β was obtained as follows:


(6) $$\text{b}={\left({\text{y}}_{1}^{T}\,{\text{y}}_{1}\right)}^{-1}\,{\text{y}}_{1}^{T}\,{\text{y}}_{2}$$

To test this estimation, the squared error between the estimated and actual values was determined:


(7) $$\text{R}\,\left(\Omega \right)={\left({\text{y}}_{2}-{\text{y}}_{1}\,\text{b}\right)}^{T}\,\left({\text{y}}_{2}-{\text{y}}_{1}\,\text{b}\right)$$

We examined the significance of specific effects to see how significantly the model parameter β explained the actual variation. This is tested with the *t* statistic using contrast *c* = [1 0 0…] of the parameter estimates *b*. The significance of a particular linear compound of effects is tested with:


(8) t=cb/ε,

where:


(9) ε2=(R⁢(Ω)/r)⁢c⁢(y1T⁢y1)-1⁢cT.

Where the degree of freedom of *t* is *r*:


(10) $$\text{r}=\text{N}-\text{rank}\,\left({\text{y}}_{1}\right)$$

*N* is the number of component data of *y_2_*. In this way, the *t*-values obtained by calculating the GLM using data of the same component among each subject were used as the ISC values. We followed previous research (Dmochowski et al., 2014) and defined the ISC value as the sum of the first through third principal components.

Finally, we performed clustering of brain activity components to examine the relationship between the brain activity components common to the subjects and the factors in the subjects’ evaluation of the music and the characteristics of the music. Based on the three-dimensional map with the first three principal components extracted by CorrCA (x-axis: first principal component, y-axis: second principal component, z-axis: third principal component), we conducted clustering for all 21 pieces of music using the k-means algorithm (MacQueen, 1967). The process of the k-means method is described as follows: (i) randomly assign a cluster to each point, (ii) calculate the center of gravity for the points assigned to each cluster, (iii) calculate the distance from the center of gravity calculated in (ii) for each point and reassign it to the cluster with the closest distance, (iv) process (ii) and (iii) until the clusters to be assigned stop changing. The process is complete when the clusters no longer change and converge. The number of clusters was determined by the elbow method and silhouette analysis to find the appropriate number of clusters.

In short, EEGs were measured while 17 participants (not selected by musical training or ability and not professional musicians) listened to 21 pieces of music selected from the 2017 Billboard Japan Hot 100, which was used as a measure of preferences reflecting engagement or interest of a large population. Participants were also asked to rate each piece of music on an 11-point scale from −5 to 5 for subjective evaluations (preference, enjoyment frequency of listening, and arousal level). ISC analysis was then performed on the acquired data using the first to third components of the EEG extracted by CorrCA, and ISC values were calculated. Then, a *t*-test was conducted on the ISC values of 10 higher-ranked and 10 lower-ranked pieces of music to examine whether music with high preferences that reflected the engagement and interest of large population had higher ISC values. In addition, a cluster analysis was conducted on all 21 pieces of music using the first three principal components of EEG as the feature values in three clusters. Then, a one-way factorial analysis of variance (ANOVA) and Tukey’s honest significant difference (HSD) *post-hoc* test were performed on the EEG principal component values and the subject’s subjective evaluation for each cluster. We also analyzed each clustered music piece’s characteristics (tonality and tempo). By doing so, we investigated which emotional and musical characteristics influenced the ISC values.

## 3. Results

**FIGURE 1:**
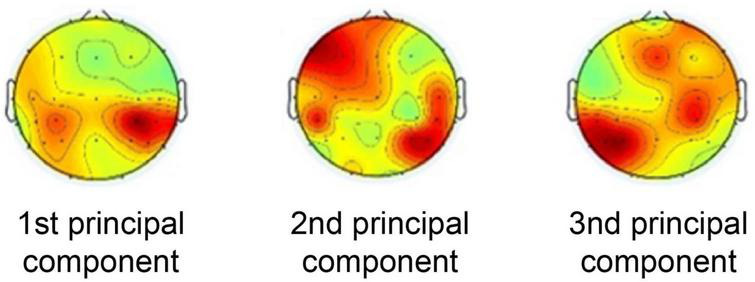
Weighting maps of the first three principal components extracted by correlated component analysis.

The weighting maps of the first three principal components extracted by CorrCA are shown in Figure 1. These are the top three principal components out of 30 that are highly correlated across subjects, indicating the weight of the common brain activity components across subjects.

**FIGURE 2:**
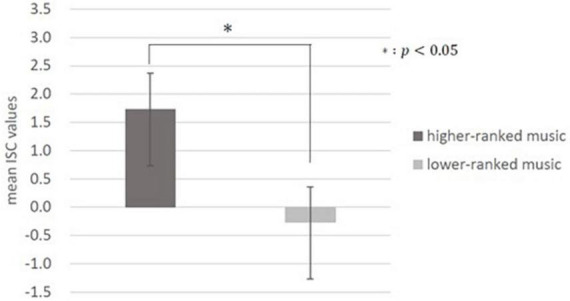
Inter-subject correlation (ISC) values for higher- and lower-ranked music on the chart. There was a significant difference between the mean ISC values of the higher-ranked music and the mean values of the lower-ranked music (*p* < 0.05). There were 10 pieces of music in the higher-ranked category and lower-ranked category, respectively.

Inter-subject correlation analysis was performed on the data calculated using the weights described above. As mentioned earlier, it has been suggested that commercials with high preference of large population have higher values of ISCs (Dmochowski et al., 2014); thus, we assumed that songs with high preference by a large population (higher-ranked music) would also have higher ISC values. In addition, one previous study conducted *t*-tests on the surprise evoked by higher-ranked versus lower-ranked music on the Billboard chart to examine whether music with higher preference by a large population evokes greater surprise (Miles et al., 2017). In the present study, to investigate whether music with high preference reflecting engagement or interest of a large population has a high ISC value, a *t*-test was conducted on the ISC values of the 10 higher-ranked and 10 lower-ranked pieces of music. As a result, there was a significant difference between the mean ISC values of the 10 higher-ranked pieces of music (1.73 ± 10.4, mean ± SD) and the mean ISC values of the 10 lower-ranked pieces of music (−0.27 ± 10.3, mean ± SD) [*t*(542) = −1.97, *p* = 0.0025; Figure 2].

In terms of the subjects’ evaluation of the preference, enjoyment, frequency of listening, and arousal of each piece of music, there was no significant difference in all items between the means of the 10 higher-ranked and 10 lower-ranked pieces of music [preference: *t*(18) = −0.164, *p* = 0.436; enjoyment: *t*(18) = −1.127, *p* = 0.863; frequency of listening: *t*(18) = −0.899, *p* = 0.19; arousal: *t*(18) = −0.215, *p* = 0.416; Supplementary Figure 1].

We also investigated whether there was a relationship between subjects’ evaluation of each piece of music and ISC values. At first, all pieces of music were re-ranked based on the average of the values of music evaluations (preference, enjoyment, frequency of listening, and arousal) of all subjects. Then, the mean ISC values of the 10 higher-ranked and 10 lower-ranked pieces of music were calculated according to that ranking. There was no significant difference between them in all items [preference: *t*(542) = −0.551, *p* = 0.291; enjoyment: *t*(542) = 1.524, *p* = 0.936; frequency of listening: *t*(542) = 0.842, *p* = 0.8; arousal: *t*(542) = 2.788, *p* = 0.997; Supplementary Figure 2].

These results suggest that ISC values may reflect preferences reflecting engagement or interest of a large population rather than subject preferences.

**FIGURE 3:**
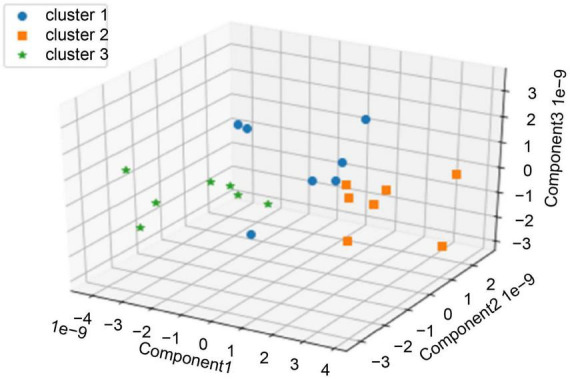
Clustering by the values of each piece of music’s top three principal components for all 21 pieces of music.

We performed clustering using the values of the first three principal components extracted from CorrCA with three clusters determined by the elbow method and silhouette analysis (Figure 3). Seven pieces of music were categorized in each cluster (Supplementary Table 2). We then examined the characteristics of each cluster, focusing on three features: values of each principal component, subjects’ evaluation, and music features. One-way factorial ANOVA and Tukey’s HSD *post-hoc* test were used to analyze the characteristics of the clusters of each principal component and subjects’ evaluation.

**FIGURE 4:**
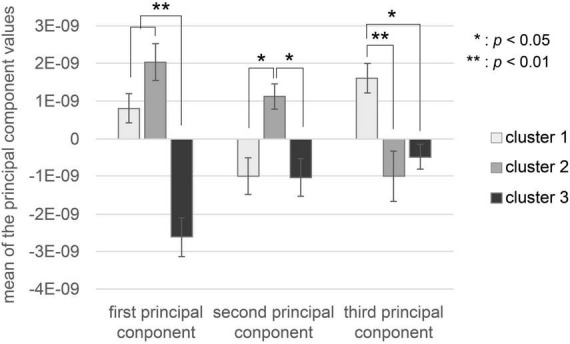
Values of each cluster’s first three principal components. The white bar represents the mean of each principal component value of music in Cluster 1. The gray bar represents the mean of each principal component value of music in Cluster 2. The black bar represents the mean of each principal component value of music in Cluster 3. Error bars represent the standard error.

The characteristics of the brain activity of each cluster are shown in Figure 4. There was a significant difference in the first principal component values in the three clusters, as shown by one-way factorial ANOVA between the three clusters [*F*(2,20) = 32.0, *p* = 0.000001]. Tukey’s HSD *post-hoc* test analysis showed that the first principal component value in Cluster 3 was significantly lower than those in Cluster 1 and Cluster 2 (Cluster 1-Cluster 3: *p* < 0.01, Cluster 2-Cluster 3: *p* < 0.01). There was a significant difference in second principal component values in the three clusters, as shown by one-way factorial ANOVA between the three clusters [*F*(2,20) = 5.689, *p* = 0.00122]. Tukey’s HSD *post-hoc* test analysis showed that the second principal component value in Cluster 2 was significantly higher than those in Cluster 1 and Cluster 3 (Cluster 1-Cluster 2: *p* < 0.05, Cluster 2-Cluster 3: *p* < 0.05). There was a significant difference in third principal component values in the three clusters, as shown by one-way factorial ANOVA between the three clusters [*F*(2,20) = 9.18, *p* = 0.00178]. The results Tukey’s HSD *post-hoc* test showed that the third principal component value in Cluster 1 was significantly higher than that in Cluster 2 and Cluster 3 (Cluster 1-Cluster 2: *p* < 0.05, Cluster 1-Cluster 3: *p* < 0.05).

**FIGURE 5:**
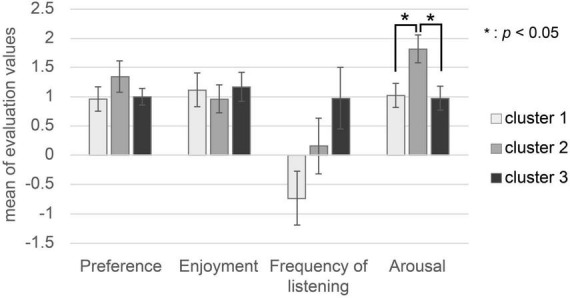
Subjects’ evaluation values (preference, enjoyment, frequency of listening, arousal). The white bar represents the mean of each subject’s evaluation value of music in Cluster 1. The gray bar represents the mean of each subject’s evaluation value of music in Cluster 2. The black bar represents the mean of each subject’s evaluation value of music in Cluster 3.

The subjects’ evaluation characteristics are shown in Figure 5. There was no significant difference in the value of preference or enjoyment in the three clusters, as shown by one-way factorial ANOVA [preference: *F*(2,50) = 1.432, *p* = 0.254; enjoyment: *F*(2,50) = 0.307, *p* = 0.738]. There was a significant difference in the value of frequency of listening in the three clusters, as shown by one-way factorial ANOVA between the three clusters [*F*(2,50) = 8.166, *p* = 0.0014]. The results of Tukey’s HSD *post-hoc* test showed that there was no significant difference in the value of frequency of listening between Cluster 1 and Cluster 2 (Cluster 1-Cluster 2: *p* = 0.41), Cluster 1 and Cluster 3 (Cluster 1-Cluster 3: *p* = 0.688), or Cluster 2 and Cluster 3 (Cluster 2-Cluster 3: *p* = 0.102). There was a significant difference in the arousal value in the three clusters, as shown by one-way factorial ANOVA between the three clusters [*F*(2,50) = 10.965, *p* = 0.0002]. Tukey’s HSD *post-hoc* test analysis showed that the arousal value in Cluster 2 was significantly higher than in Cluster 1 and Cluster 3 (Cluster 1-Cluster 2: *p* < 0.05, Cluster 2-Cluster 3: *p* < 0.05).

The characteristics of the music are shown in Table 1. Mean tempo values did not differ significantly in the three clusters, as shown by one-way factorial ANOVA between the three clusters [*F*(2, 20) = 2.250, *p* = 0.134]. However, Cluster 1 and Cluster 3 had a faster tempo, while Cluster 2 had a slower tempo than did Cluster 1 and Cluster 3. In terms of tonality, Cluster 1 had music predominantly in a major key, while Cluster 3 had music predominantly in a minor key. In addition, Cluster 1 had only pop music, while Cluster 2 had many ballad-like pieces of music (Supplementary Table 2).

**TABLE 1:** Music characteristics.

	Tonality		Tempo [mean ± standard deviation (SD)]
	Major key	Minor key	
Cluster 1	6	1	131.6 ± 31.4
Cluster 2	4	3	105.4 ± 25.2
Cluster 3	2	5	144.1 ± 38.8

Mean tempo values did not differ significantly between clusters [*F*(2,20) = 2.250, *p* = 0.134]. However, Cluster 2 had a slower tempo than did Cluster 1 and Cluster 3. In terms of tonality, Cluster 1 had music predominantly in the major key, while Cluster 3 had music predominantly in the minor key.

These results indicate that brain activity, subjects’ evaluations, and music characteristics are present in each cluster. Additionally, the principal components that correlate well across subjects may represent the subjects’ arousal level and the characteristics of the music (tonality and tempo).

## 4. Discussion

In this study, we investigated whether the ISCs of EEGs during music listening represents a preference for music reflecting engagement or interest of a large population in music. The results showed that the ISC values of the higher-ranked music, which are considered to have high preferences reflecting engagement or interest of a large population, were significantly higher than those of lower-ranked music (Figure 2). By contrast, there was no significant difference in ISC values between the higher- and lower-ranked music based on subjects’ subjective evaluation of their preferences (Supplementary Figure 2). This suggests that ISC values reflect the preferences reflecting engagement or interest of a large population rather than the subjects’ preferences. This is consistent with the results of Dmochowski et al. (2014), who suggested that ISC values could predict the preferences of large audiences rather than subjects’ own preferences for commercials. This study showed similar results for music rather than videos such as commercials.

One reason for this finding is that subjective evaluations of music vary widely among individuals, an aspect that has fostered musical diversity. On the contrary, brain activity reflects unconscious information processing. It is thought that collectively shared aspects of music information processing can be extracted, although they do not necessarily correspond to subjective evaluation. Previous studies found that self-reports of ratings of music are influenced by bias (Rosenman et al., 2011) and limited by listeners’ ability to self-evaluate (Madsen et al., 1993), but, like neural responses, time-changing ISC measures of engagement suggest that engagement during music listening can be objectively indexed (Kaneshiro et al., 2021).

It has been suggested that ISCs during music listening may be driven by melodic and tonal expectation formation (facing and processing novel content) and expectation processing (especially expectation violation) (Kaneshiro et al., 2020). Additionally, it has been suggested that viewers’ ISCs while watching a movie reflect engagement (Dmochowski et al., 2012), emotional arousal (Nummenmaa et al., 2012), and immersion in the movie (Cohen et al., 2017). Dmochowski et al. (2014) found that top-down modulation, in which individual preferences may alter the person’s attention and engagement, may affect the strength of neural responses associated with stimulus-locked neural processing and ISC. However, if individual preferences guide the modulation of sensory processing, it may be that the ISC will reflect the subject’s preference rather than the group’s preference. However, an individual’s preference for a stimulus may be strongly driven by the narrative that stimulus in the brain and such bottom-up influences well reflect preferences of large population. Nonetheless, this sensory processing may be masked by individual preferences and biases (Dmochowski et al., 2014). This means that the results of the present study, may indicating that ISC values reflect preference reflecting engagement or interest of a large population rather than subject preferences, suggest that the ISC reflects subjects’ unconscious, inward sensory processing influenced by the narrative nature of the music (bottom-up). Accordingly, as brain activity during music listening (ISC values) may represent unconscious responses (emotions) shared by the large population, no relationship was found between ISC values and subjects’ subjective evaluations.

In addition, Dmochowski et al. (2014) used ratings of preferences for commercials by over 7,000 participants as an indicator of preferences of large populations. However, in this study, we used the Billboard Japan Hot 100 chart as an indicator of preference reflecting engagement or interest, which is a composite index that refers not only to CD sales and music downloads, but also to video views on YouTube and other media and the number of tweets mentioning the song and artist name. Thus, we cannot assume with certainty that people purchased or played the song or tweeted the song and the artist’s name because they necessarily liked the song. Furthermore, based on our observations above, as ISC values may represent unconscious responses, it is more appropriate to interpret this result as simply indicating interest, rather than that ISC values represent preference reflecting engagement or interest of a large population.

Therefore, these ISC values can be considered to represent the interest of a large population in music, which is subconsciously aroused in people by music listening.

We also investigated which emotional and musical characteristics influenced the ISCs of EEGs during music listening. Specifically, a cluster analysis was conducted using the EEG correlation components between subjects as features, which were used to calculate ISC values. The results showed that brain activity, subjects’ evaluations, and music features were present in each cluster (Figures 3–5; Table 1).

First, Cluster 1 was characterized by subjects’ infrequent listening and frequent use of music in a major key, with particularly high values for the third principal component. A previous study conjectured that major chords, frequently used in music in major keys, induce significant activity in the left middle temporal gyrus (Suzuki et al., 2008). Therefore, these results indicate that listening to music in major keys increased the activity of the left middle temporal gyrus, which is prominent in the third principal component.

Cluster 2 was characterized by subjects’ high preference, especially high arousal, and relatively slow-tempo balladic music, with particularly high values for the second principal component. Previous studies have suggested that the amygdala is more active when listening to pleasant music (Mueller et al., 2011; Koelsch et al., 2013). It has also been suggested that the right temporal lobe is more active and the right frontal lobe is less active when listening to emotional music (Alfredson et al., 2004). Therefore, given the strong influence of the second principal component in this result, it is assumed that the activity of the OFC, which is influenced by the activity of the amygdala, became more active when the subjects listened to highly favorable music. In addition, listening to sentimental, relatively slow-tempo music that uplifted the subject’s mood enhanced activity in the right temporal lobe and suppressed activity in the right frontal lobe.

Finally, Cluster 3 was characterized by subjects’ high listening frequency and frequent use of music in minor keys, with particularly low values for the first principal component (and low values for the second and third principal components). Previous studies have suggested that minor chords, often used in music in minor keys, show significant activity in the right striatum (Suzuki et al., 2008) and that the amygdala, vastus posterior cortex, brainstem, and cerebellum are more active compared to major chords (Pallesen et al., 2005). However, the results suggest that listening to music in a minor key did not affect activity in the aforementioned brain regions.

In this study, music with lyrics was utilized as the stimulus due to the use of a Billboard chart as a measure of preference reflecting engagement or interest of a large population. Previously, Brattico et al. (2011) investigated the impact of lyrics on emotional processing of music using fMRI to measure brain responses while subjects listened to happy music (featuring a high usage of major chords) and sad music (featuring a low usage of major chords). The findings revealed that sad music with lyrics activated brain regions such as the parahippocampal gyrus, amygdala, claustrum, putamen, precentral gyrus, medial and IFG, and auditory cortex to a greater extent compared to sad music without lyrics. Additionally, happy music without lyrics activated the limbic system and the right pars opercularis of the IFG, whereas happy music with lyrics primarily elicited responses in the auditory cortex. Behavioral assessments also indicated that happy music without lyrics evoked stronger positive emotions than happy music with lyrics. These findings suggest that lyrics may indeed influence emotions and brain responses during music listening.

However, it is important to note that although the results of the current study revealed that Cluster 1 was characterized by frequent use of music in major keys and particularly high values for the third principal component while Cluster 3 was characterized by frequent use of music in minor keys and particularly low values for the first principal component (as well as low values for the second and third principal components), considering the previous studies mentioned above, it cannot be definitively concluded that lyrics had an influence in this particular analysis.

From these findings, the principal components with high correlation among subjects used to calculate the ISC values are considered to represent the subjects’ arousal level and the characteristics of the music (tonality and tempo), influencing ISC values.

Previous studies have investigated the relationship between the ISCs of EEGs and the stimulus-response correlation (SRC) while subjects were experiencing audiovisual stimuli (Dmochowski et al., 2018). SRC represents the extent to which temporally varying stimulus features are correlated with the evoked EEG response and has been calculated to measure how strongly a stimulus drives individual neural responses; both ISC and SRC have been suggested to increase with heightened attention and to decrease with reduced attention (Ki et al., 2016, 2020; Dmochowski et al., 2018). In addition, Dmochowski et al. (2012) showed that peak values of neural correlates during movie viewing corresponded significantly with the exciting moments of the movie, such as scenes in which the protagonist is holding a gun (highly suspenseful, tense, and surprising) and tense scenes in movies related to hands. Moreover, Nummenmaa et al. (2012) conjectured that subjects’ arousal ratings during movie viewing positively correlated with ISC in the visual cortex, somatosensory cortex, bilateral intraparietal sulcus, and frontal eye area. They suggested that ISC reflects emotional arousal to narrative stimuli (Dmochowski et al., 2012; Nummenmaa et al., 2012). Therefore, previous studies also suggest that ISC values are affected by arousal levels.

An fMRI study to examine the neural basis of acoustic features of naturalistic music stimuli revealed that tonal and rhythmic components showed significant ISCs (Alluri et al., 2012). Besides, Kaneshiro et al. (2020) researched ISCs and SRC while listening to original musical stimuli that retained basic musical features such as rhythm and melody and phase-scrambled and time-scrambled (measure shuffle) stimuli. Results have shown that SRC correlates with ISC (Dmochowski et al., 2018), and both ISC and SRC are significantly correlated at beat-related frequencies (Kaneshiro et al., 2020). Therefore, previous studies suggest that the characteristics of music influence ISC values.

To summarize, it is suggested that the ISC values in this study may represent the subconsciously aroused interest of a large population in music. Furthermore, the principal components highly correlated across subjects used to calculate the ISC values represent the subjects’ arousal level and characteristics of the music, suggesting that they influence the ISC values. Thus, the use of charts that refer not only to the extent to which people purchase music, but also to their reactions to music in online networks, and the selection of songs from that chart as a way of evaluating behavioral responses of large populations toward music. Hence, our study adds novelty to the field of study regarding ISC engagement in that ISC engagement may represent “the interest aroused in large population, influenced by subjects’ arousal level and characteristics of the music.”

To establish the relationships observed in this study, we initially speculated based on previous research findings. First, studies have indicated that specific acoustic features of music, such as timbre, tempo, and dissonance, are associated with particular emotional responses like anger, happiness, and arousal (Juslin and Laukka, 2000; Gabrielson and Juslin, 2003; Koelsch et al., 2006; Gomez and Danuser, 2007; Trost et al., 2015). Therefore, it is possible that the acoustic features of the music used in this study induced arousal in the subjects and influenced the calculated ISC values. Additionally, as previous research suggests that repeated exposure to music increases preference (Madison and Schiölde, 2017), it is plausible that more frequent listening to music subconsciously increased interest, which could be reflected in the ISC values. However, the results of this study did not indicate a direct relationship between the principal components that exhibited high correlation across subjects and the frequency of listening. Hence, it cannot be concluded that listening frequency directly affected the interest in music of large population or ISC values. Consequently, we inferred from the results of this study that ISC values might be closely associated with the experience of musical pleasure resulting from prediction errors. The underlying reasons for this inference are discussed below.

Kaneshiro et al. (2020) also showed that while the unedited original stimuli were subjectively evaluated as the most pleasant, the highest ISC values were found for the time-scrambled (measure-shuffled) stimuli. This result is thought to represent a situation in which the measure-level beat structure strongly evokes a sense of temporal cohesion. At the same time, the lack of contextual connection between measures subtly betrays expectations of melody and tonality and increases listening attention (Kaneshiro et al., 2020). Thus, it has been suggested that ISCs during music listening may be driven by expectation formation (facing and processing novel content) and expectation processing (especially expectation violation) (Kaneshiro et al., 2020).

Additionally, Madsen et al. (2019) interpreted the slope of ISC change across repeats during repeated listening to music as the persistence of interest in the music. The results of our study did not indicate that frequency of music listening affected interest in music, but as discussed below, this may be because some songs increase ISC values with repeated listening, while others decrease ISC values. Madsen et al. (2019) showed that music with increasing ISC values contains large changes in volume and introduces new instruments and timbres, and flourishes as the passage progresses, avoiding the predictability that occurs in many other pieces of music. In contrast, music with steeper ISC drops also contains large volume changes, but the same patterns are often repeated, making the music predictable. It has been suggested that the reason for this is that as subjects listen to the same patterns over and over again, the surprise effect disappears, and the ISC may decrease as the EEG response (P300, mismatch negativity, error-related potentials) (Escera and Corral, 2007), which is thought to indicate novelty, decreases (Madsen et al., 2019).

Furthermore, recent studies suggest that predictive mechanisms may drive musical pleasure (Salimpoor et al., 2015; Gold et al., 2019; Koelsch et al., 2019). It has been suggested that uncertainty when predicting how the next acoustic feature will change during listening to music and the surprise response when the music actually deviates from the prediction activates the brain’s reward system and causes pleasant sensations (Cheung et al., 2019; Leahy et al., 2021).

The results of this study suggest that subjects’ arousal values during music listening and music characteristics affect ISC values and that ISC values may represent the interest in music of large population. Previous studies have suggested that ISCs during music listening may be driven by expectation formation and expectation violation due to changes in music features (Kaneshiro et al., 2020) and may be related to interest in music and surprise effects (Madsen et al., 2019). Consequently, the results of this study, together with the findings in previous studies about musical pleasure and predictive mechanisms (Salimpoor et al., 2015; Cheung et al., 2019; Gold et al., 2019; Koelsch et al., 2019; Leahy et al., 2021), suggest that ISC values are closely related to musical pleasantness due to prediction error.

Several limitations of this study need to be considered. First, the use of music with lyrics for experimental stimuli introduced potential confounding (Brattico et al., 2011) and could have influenced the results. As noted earlier, this study’s results do not indicate any influence from lyrics. However, several previous studies, such as Madsen et al. (2019), avoided this confounding by using instrumental music as stimuli, and Kaneshiro et al. (2020) used music whose lyrics were composed in Hindi as stimuli, which were difficult for participants to understand, thus avoiding confounding. Therefore, for future studies, it will be necessary to utilize stimuli such as music without lyrics, music with lyrics in a foreign language that is difficult for participants to understand, or music in which only the singing voice is replaced with instrumental sounds using music production and editing software. This approach will help isolate the influence of lyrics on the observed effects. Second, while this study focused on analyzing tonality and tempo as acoustic features of the music and demonstrating their influence on ISC, further analysis is needed to explore the impact of other acoustic features on ISC. Given the use of primarily pop songs in this study, future research should consider using a variety of music genres as stimuli, as acoustic features are likely to differ across different types of music. Detailed analysis of acoustic features using MATLAB’s MIRtoolbox can provide further insights into the influence of these features on ISC. Third, as ISC values were calculated and examined for each piece of music during the listening session, it remains unclear how ISC values change over time within a specific piece of music. Therefore, in future studies, analyzing ISC values for multiple musical stimuli in time-series data would allow for the exploration of the temporal dynamics of ISC and its relationship with acoustic features and emotional responses. By addressing these considerations in future research, we can further enhance our understanding of the influence of different stimuli, acoustic features, and temporal dynamics on ISC and its relation to emotional processing during music listening.

Future studies combining these proposed methods will shed more light on how ISC reflects the unconsciously aroused interest in music under the combined influence of arousal level and acoustic features, as well as on the mechanisms by which music induces pleasure.

## 5. Conclusion

This study aimed to clarify whether the ISCs of EEGs during music listening represent music preferences reflecting engagement or interest of a large population. Our results suggest that the ISC values calculated represent the subconscious interest in music of a large population. Furthermore, the principal components, highly correlated across subjects, used to calculate ISC values represent subjects’ arousal levels and characteristics of the music. Hence, the findings of this study suggest that subjects’ arousal values during music listening, as well as the specific characteristics of the music itself, influence the calculated ISC values. Furthermore, ISC values may serve as a representation of the interest in music of a large population.

## Data availability statement

The raw data supporting the conclusions of this article will be made available by the authors, without undue reservation.

## Ethics statement

The studies involving humans were approved by the Ethics Committee of the School of Science and Technology, Meiji University. The studies were conducted in accordance with the local legislation and institutional requirements. The participants provided their written informed consent to participate in this study.

## Author contributions

FU and SS designed the experiments and wrote the manuscript. FU performed the experiments, collected the data, and analyzed the data. Both authors contributed to the article and approved the submitted version.

## Footnotes

1
https://www.audacityteam.org/

## References

[B1] Abrams D. A. Ryali S. Chen T. Chordia P. Khouzam A. Levitin D. J. (2013). Inter-subject synchronization of brain responses during natural music listening. *Eur. J. Neurosci.* 37 1458–1469. 10.1111/ejn.12173 23578016 PMC4487043

[B2] Alfredson B. B. Risberg J. Hagberg B. Gustafson L. (2004). Right temporal lobe activation when listening to emotionally significant music. *Appl. Neuropsychol.* 11 161–166. 10.1207/s15324826an1103_4 15590350

[B3] Alluri V. Toiviainen P. Jääskeläinen I. P. Glerean E. Sams M. Brattico E. (2012). Large-scale brain networks emerge from dynamic processing of musical timbre, key and rhythm. *Neuroimage* 59 3677–3689. 10.1016/j.neuroimage.2011.11.019 22116038

[B4] Ara A. Marco-Pallarés J. (2020). Fronto-temporal theta phase-synchronization underlies music-evoked pleasantness. *Neuroimage* 212:116665. 10.1016/j.neuroimage.2020.116665 32087373

[B5] Blood A. J. Zatorre R. J. (2001). Intensely pleasurable responses to music correlate with activity in brain regions implicated in reward and emotion. *Proc. Natl. Acad. Sci. U.S.A.* 98 11818–11823. 10.1073/pnas.191355898 11573015 PMC58814

[B6] Bolton T. A. W. Freitas L. G. A. Jochaut D. Giraud A. L. Van De Ville D. (2020). Neural responses in autism during movie watching: Inter-individual response variability co-varies with symptomatology. *Neuroimage* 216:116571. 10.1016/j.neuroimage.2020.116571 31987996

[B7] Brattico E. Alluri V. Bogert B. Jacobsen T. Vartiainen N. Nieminen S. (2011). A functional MRI study of happy and sad emotions in music with and without lyrics. *Front. Psychol.* 2:308. 10.3389/fpsyg.2011.00308 22144968 PMC3227856

[B8] Cantlon J. F. Li R. (2013). Neural activity during natural viewing of Sesame Street statistically predicts test scores in early childhood. *PLoS Biol.* 11:e1001462. 10.1371/journal.pbio.1001462 23300385 PMC3536813

[B9] Chen J. L. Penhune V. B. Zatorre R. J. (2008). Listening to musical rhythms recruits motor regions of the brain. *Cereb. Cortex* 18 2844–2854. 10.1093/cercor/bhn042 18388350

[B10] Chen Y. Farivar R. (2020). Natural scene representations in the gamma band are prototypical across subjects. *Neuroimage* 221 117010. 10.1016/j.neuroimage.2020.117010 32505697

[B11] Cheung V. K. M. Harrison P. M. C. Meyer L. Pearce M. T. Haynes J. D. Koelsch S. (2019). Uncertainty and surprise jointly predict musical pleasure and amygdala, hippocampus, and auditory cortex activity. *Curr. Biol.* 29 4084–4092.e4. 10.1016/j.cub.2019.09.067 31708393

[B12] Cohen S. S. Henin S. Parra L. C. (2017). Engaging narratives evoke similar neural activity and lead to similar time perception. *Sci. Rep.* 7:4578. 10.1038/s41598-017-04402-4 28676688 PMC5496904

[B13] Cohen S. S. Tottenham N. Baldassano C. (2022). Developmental changes in story-evoked responses in the neocortex and hippocampus. *Elife* 11:e69430. 10.7554/eLife.69430 35787304 PMC9328767

[B14] Daly I. Williams D. Hwang F. Kirke A. Miranda E. R. Nasuto S. J. (2019). Electroencephalography reflects the activity of sub-cortical brain regions during approach-withdrawal behaviour while listening to music. *Sci. Rep.* 9:9415. 10.1038/s41598-019-45105-2 31263113 PMC6603018

[B15] Dauer T. Nguyen D. T. Gang N. Dmochowski J. P. Berger J. Kaneshiro B. (2021). Inter-subject correlation while listening to minimalist music: A study of electrophysiological and behavioral responses to Steve Reich’s Piano Phase. *Front. Neurosci.* 15:702067. 10.3389/fnins.2021.702067 34955706 PMC8695499

[B16] Dmochowski J. P. Bezdek M. A. Abelson B. P. Johnson J. S. Schumacher E. H. Parra L. C. (2014). Audience preferences are predicted by temporal reliability of neural processing. *Nat. Commun.* 5:4567. 10.1038/ncomms5567 25072833 PMC4124862

[B17] Dmochowski J. P. Ki J. J. DeGuzman P. Sajda P. Parra L. C. (2018). Extracting multidimensional stimulus-response correlations using hybrid encoding-decoding of neural activity. *Neuroimage* 180 134–146. 10.1016/j.neuroimage.2017.05.037 28545933

[B18] Dmochowski J. P. Sajda P. Dias J. Parra L. C. (2012). Correlated components of ongoing EEG point to emotionally laden attention - a possible marker of engagement? *Front. Hum. Neurosci.* 6:112. 10.3389/fnhum.2012.00112 22623915 PMC3353265

[B19] Escera C. Corral M. J. (2007). Role of mismatch negativity and novelty-P3 in involuntary auditory attention. *J. Psychophysiol.* 21 251–264. 10.1027/0269-8803.21.34.251

[B20] Farbood M. M. Heeger D. J. Marcus G. Hasson U. Lerner Y. (2015). The neural processing of hierarchical structure in music and speech at different timescales. *Front. Neurosci.* 9:157. 10.3389/fnins.2015.00157 26029037 PMC4429236

[B21] Finn E. S. Corlett P. R. Chen G. Bandettini P. A. Constable R. T. (2018). Trait paranoia shapes inter-subject synchrony in brain activity during an ambiguous social narrative. *Nat. Commun.* 9:2043. 10.1038/s41467-018-04387-2 29795116 PMC5966466

[B22] Foster N. E. V. Halpern A. R. Zatorre R. J. (2013). Common parietal activation in musical mental transformations across pitch and time. *Neuroimage* 75 27–35. 10.1016/j.neuroimage.2013.02.044 23470983

[B23] Friston K. J. Holmes A. P. Worsley K. J. Poline J.-P. Frith C. D. Frackowiak R. S. J. (1994). Statistical parametric maps in functional imaging: A general linear approach. *Hum. Brain Mapp.* 2 189–210. 10.1002/hbm.460020402

[B24] Frühholz S. Trost W. Kotz S. A. (2016). The sound of emotions-towards a unifying neural network perspective of affective sound processing. *Neurosci. Biobehav. Rev.* 68 96–110. 10.1016/j.neubiorev.2016.05.002 27189782

[B25] Gabrielson A. Juslin P. N. (2003). “Emotional expression in music,” in *Handbook of affective sciences*, eds Davidson R. J. Scherer K. R. Goldsmith H. H. (New York, NY: Oxford University Press).

[B26] Gold B. P. Pearce M. T. Mas-Herrero E. Dagher A. Zatorre R. J. (2019). Predictability and uncertainty in the pleasure of music: A reward for learning? *J. Neurosci.* 39 9397–9409. 10.1523/JNEUROSCI.0428-19.2019 31636112 PMC6867811

[B27] Gomez P. Danuser B. (2007). Relationships between musical structure and psychophysiological measures of emotion. *Emotion* 7 377–387. 10.1037/1528-3542.7.2.377 17516815

[B28] Grahn J. A. Brett M. (2007). Rhythm and beat perception in motor areas of the brain. *J. Cogn. Neurosci.* 19 893–906. 10.1162/jocn.2007.19.5.893 17488212

[B29] Gruskin D. C. Rosenberg M. D. Holmes A. J. (2020). Relationships between depressive symptoms and brain responses during emotional movie viewing emerge in adolescence. *Neuroimage* 216:116217. 10.1016/j.neuroimage.2019.116217 31628982 PMC7958984

[B30] Hasson U. Landesman O. Knappmeyer B. Vallines I. Rubin N. Heeger D. J. (2008). Neurocinematics: The neuroscience of film. *Projections* 2 1–26. 10.3167/proj.2008.020102

[B31] Hasson U. Nir Y. Levy I. Fuhrmann G. Malach R. (2004). Intersubject synchronization of cortical activity during natural vision. *Science* 303 1634–1640. 10.1126/science.1089506 15016991

[B32] Hotelling H. (1936). Relations between two sets of variates. *Biometrika* 28 321–377. 10.1093/biomet/28.3-4.321

[B33] Janata P. Birk J. L. Van Horn J. D. Leman M. Tillmann B. Bharucha J. J. (2002). The cortical topography of tonal structures underlying Western music. *Science* 298 2167–2170. 10.1126/science.1076262 12481131

[B34] Jäncke L. Alahmadi N. (2016). Detection of independent functional networks during music listening using electroencephalogram and sLORETA-ICA. *Neuroreport* 27 455–461. 10.1097/WNR.0000000000000563 26934285

[B35] Juslin P. N. Laukka P. (2000). Improving emotional communication in music performance through cognitive feedback. *Musicae Sci.* 4 151–183.

[B36] Kandeepan S. Rudas J. Gomez F. Stojanoski B. Valluri S. Owen A. M. (2020). Modeling an auditory stimulated brain under altered states of consciousness using the generalized Ising model. *Neuroimage* 223:117367. 10.1016/j.neuroimage.2020.117367 32931944

[B37] Kaneshiro B. Nguyen D. T. Norcia A. M. Dmochowski J. P. Berger J. (2020). Natural music evokes correlated EEG responses reflecting temporal structure and beat. *Neuroimage* 214:116559. 10.1016/j.neuroimage.2020.116559 31978543

[B38] Kaneshiro B. Nguyen D. T. Norcia A. M. Dmochowski J. P. Berger J. (2021). Inter-subject EEG correlation reflects time-varying engagement with natural music. *bioRxiv* [Preprint]. 10.1101/2021.04.14.439913 38626924

[B39] Kauppi J. P. Jääskeläinen I. P. Sams M. Tohka J. (2010). Inter-subject correlation of brain hemodynamic responses during watching a movie: Localization in space and frequency. *Front. Neuroinform.* 4:5. 10.3389/fninf.2010.00005 20428497 PMC2859808

[B40] Ki J. J. Kelly S. P. Parra L. C. (2016). Attention strongly modulates reliability of neural responses to naturalistic narrative stimuli. *J. Neurosci.* 36 3092–3101. 10.1523/JNEUROSCI.2942-15.2016 26961961 PMC6601758

[B41] Ki J. J. Parra L. C. Dmochowski J. P. (2020). Visually evoked responses are enhanced when engaging in a video game. *Eur. J. Neurosci.* 52 4695–4708. 10.1111/ejn.14924 32735746 PMC7818444

[B42] Koelsch S. (2020). A coordinate-based meta-analysis of music-evoked emotions. *Neuroimage* 223:117350. 10.1016/j.neuroimage.2020.117350 32898679

[B43] Koelsch S. Fritz T. Cramon D. Y. V. Müller K. Friederici A. D. (2006). Investigating emotion with music: An fMRI study. *Hum. Brain Mapp.* 27 239–250. 10.1002/hbm.20180 16078183 PMC6871371

[B44] Koelsch S. Skouras S. Fritz T. Herrera P. Bonhage C. Küssner M. B. (2013). The roles of superficial amygdala and auditory cortex in music-evoked fear and joy. *Neuroimage* 81 49–60. 10.1016/j.neuroimage.2013.05.008 23684870

[B45] Koelsch S. Vuust P. Friston K. (2019). Predictive processes and the peculiar case of music. *Trends Cogn. Sci.* 23 63–77. 10.1016/j.tics.2018.10.006 30471869

[B46] Kotila A. Tohka J. Kauppi J. P. Gabbatore I. Mäkinen L. Hurtig T. M. (2021). Neural-level associations of non-verbal pragmatic comprehension in young Finnish autistic adults. *Int. J. Circumpolar. Health* 80:1909333. 10.1080/22423982.2021.1909333 34027832 PMC8158210

[B47] Laird A. R. Robinson J. L. McMillan K. M. Tordesillas-Gutiérrez D. Moran S. T. Gonzales S. M. (2010). Comparison of the disparity between Talairach and MNI coordinates in functional neuroimaging data: Validation of the Lancaster transform. *Neuroimage* 51 677–683. 10.1016/j.neuroimage.2010.02.048 20197097 PMC2856713

[B48] Leahy J. Kim S. G. Wan J. Overath T. (2021). An analytical framework of tonal and rhythmic hierarchy in natural music using the multivariate temporal response function. *Front. Neurosci.* 15:665767. 10.3389/fnins.2021.665767 34335154 PMC8322238

[B49] LeDoux J. E. (2000). Emotion circuits in the brain. *Annu. Rev. Neurosci.* 23 155–184. 10.1146/annurev.neuro.23.1.155 10845062

[B50] Lerner Y. Bleich-Cohen M. Solnik-Knirsh S. Yogev-Seligmann G. Eisenstein T. Madah W. (2018). Abnormal neural hierarchy in processing of verbal information in patients with schizophrenia. *Neuroimage Clin.* 17 1047–1060. 10.1016/j.nicl.2017.12.030 29349038 PMC5768152

[B51] Liu Y. Lian W. Zhao X. Tang Q. Liu G. (2021). Spatial connectivity and temporal dynamic functional network connectivity of musical emotions evoked by dynamically changing tempo. *Front. Neurosci.* 15:700154. 10.3389/fnins.2021.700154 34421523 PMC8375772

[B52] MacQueen J. (1967). Some methods for classification and analysis of multivariate observations, in *Berkeley symposium on mathematical statistics and probability*, eds Le Cam L. M. Neyman J. (Los Angeles, CA: University of California), 281–297.

[B53] Madison G. Schiölde G. (2017). Repeated listening increases the liking for music regardless of its complexity: Implications for the appreciation and aesthetics of music. *Front. Neurosci.* 11:147. 10.3389/fnins.2017.00147 28408864 PMC5374342

[B54] Madsen C. K. Brittin R. V. Capperella-Sheldon D. A. (1993). An empirical method for measuring the aesthetic experience to music. *J. Res. Music Educ.* 41 57–69. 10.2307/3345480

[B55] Madsen J. Margulis E. H. Simchy-Gross R. Parra L. C. (2019). Music synchronizes brainwaves across listeners with strong effects of repetition, familiarity and training. *Sci. Rep.* 9:3576. 10.1038/s41598-019-40254-w 30837633 PMC6401073

[B56] Mas-Herrero E. Dagher A. Farrés-Franch M. Zatorre R. J. (2021a). Unraveling the temporal dynamics of reward signals in music-induced pleasure with TMS. *J. Neurosci.* 41 3889–3899. 10.1523/JNEUROSCI.0727-20.2020 33782048 PMC8084325

[B57] Mas-Herrero E. Maini L. Sescousse G. Zatorre R. J. (2021b). Common and distinct neural correlates of music and food-induced pleasure: A coordinate-based meta-analysis of neuroimaging studies. *Neurosci. Biobehav. Rev.* 123 61–71. 10.1016/j.neubiorev.2020.12.008 33440196

[B58] Miles S. A. Rosen D. S. Grzywacz N. M. (2017). A statistical analysis of the relationship between harmonic surprise and preference in popular music. *Front. Hum. Neurosci.* 11:263. 10.3389/fnhum.2017.00263 28572763 PMC5435755

[B59] Mueller K. Mildner T. Fritz T. Lepsien J. Schwarzbauer C. Schroeter M. L. (2011). Investigating brain response to music: A comparison of different fMRI acquisition schemes. *Neuroimage* 54 337–343. 10.1016/j.neuroimage.2010.08.029 20728550

[B60] Nummenmaa L. Glerean E. Viinikainen M. Jääskeläinen I. P. Hari R. Sams M. (2012). Emotions promote social interaction by synchronizing brain activity across individuals. *Proc. Natl. Acad. Sci. U.S.A.* 109 9599–9604. 10.1073/pnas.1206095109 22623534 PMC3386135

[B61] Ou W. Zeng W. Gao W. He J. Meng Y. Fang X. (2022). Movie events detecting reveals inter-subject synchrony difference of functional brain activity in autism spectrum disorder. *Front. Comput. Neurosci.* 16:877204. 10.3389/fncom.2022.877204 35591883 PMC9110681

[B62] Pallesen K. J. Brattico E. Bailey C. Korvenoja A. Koivisto J. Gjedde A. (2005). Emotion processing of major, minor, and dissonant chords: A functional magnetic resonance imaging study. *Ann. N. Y. Acad. Sci.* 1060 450–453. 10.1196/annals.1360.047 16597801

[B63] Pando-Naude V. Patyczek A. Bonetti L. Vuust P. (2021). An ALE meta-analytic review of top-down and bottom-up processing of music in the brain. *Sci. Rep.* 11:20813. 10.1038/s41598-021-00139-3 34675231 PMC8531391

[B64] Parra L. C. Spence C. D. Gerson A. D. Sajda P. (2005). Recipes for the linear analysis of EEG. *Neuroimage* 28 326–341. 10.1016/j.neuroimage.2005.05.032 16084117

[B65] Patel G. H. Arkin S. C. Ruiz-Betancourt D. R. Plaza F. I. Mirza S. A. Vieira D. J. (2021). Failure to engage the temporoparietal junction/posterior superior temporal sulcus predicts impaired naturalistic social cognition in schizophrenia. *Brain* 144 1898–1910. 10.1093/brain/awab081 33710282 PMC8320281

[B66] Rosenman R. Tennekoon V. Hill L. G. (2011). Measuring bias in self-reported data. *Int. J. Behav. Healthc. Res.* 2 320–332. 10.1504/ijbhr.2011.043414 25383095 PMC4224297

[B67] Royal I. Vuvan D. T. Zendel B. R. Robitaille N. Schönwiesner M. Peretz I. (2016). Activation in the right inferior parietal lobule reflects the representation of musical structure beyond simple pitch discrimination. *PLoS One* 11:e0155291. 10.1371/journal.pone.0155291 27195523 PMC4873218

[B68] Sachs M. E. Habibi A. Damasio A. Kaplan J. T. (2020). Dynamic intersubject neural synchronization reflects affective responses to sad music. *Neuroimage* 218:116512. 10.1016/j.neuroimage.2019.116512 31901418

[B69] Salimpoor V. N. van den Bosch I. Kovacevic N. McIntosh A. R. Dagher A. Zatorre R. J. (2013). Interactions between the nucleus accumbens and auditory cortices predict music reward value. *Science* 340 216–219. 10.1126/science.1231059 23580531

[B70] Salimpoor V. N. Zald D. H. Zatorre R. J. Dagher A. McIntosh A. R. (2015). Predictions and the brain: How musical sounds become rewarding. *Trends Cogn. Sci.* 19 86–91. 10.1016/j.tics.2014.12.001 25534332

[B71] Schmälzle R. Grall C. (2020). “Mediated messages and synchronized brains,” in *The handbook of communication science and biology, series*, eds Flyd K. Weber R. (New York, NY: Routledge), 109–122.

[B72] Schmälzle R. Häcker F. E. Honey C. J. Hasson U. (2015). Engaged listeners: Shared neural processing of powerful political speeches. *Soc. Cogn. Affect. Neurosci.* 10 1137–1143. 10.1093/scan/nsu168 25653012 PMC4526488

[B73] Schmälzle R. Häcker F. Renner B. Honey C. J. Schupp H. T. (2013). Neural correlates of risk perception during real-life risk communication. *J. Neurosci.* 33 10340–10347. 10.1523/JNEUROSCI.5323-12.2013 23785147 PMC3755178

[B74] Schubert E. Vincs K. Stevens C. J. (2013). Identifying regions of good agreement among responders in engagement with a piece of live dance. *Empir. Stud. Arts* 31 1–20. 10.2190/EM.31.1.a 22612255

[B75] Schulze K. Zysset S. Mueller K. Friederici A. D. Koelsch S. (2011). Neuroarchitecture of verbal and tonal working memory in nonmusicians and musicians. *Hum. Brain Mapp.* 32 771–783. 10.1002/hbm.21060 20533560 PMC6870416

[B76] Sihvonen A. J. Särkämö T. (2022). Music processing and amusia. *Handb. Clin. Neurol.* 187 55–67. 10.1016/b978-0-12-823493-8.00014-6 35964992

[B77] Simony E. Honey C. J. Chen J. Lositsky O. Yeshurun Y. Wiesel A. (2016). Dynamic reconfiguration of the default mode network during narrative comprehension. *Nat. Commun.* 7:12141. 10.1038/ncomms12141 27424918 PMC4960303

[B78] Suzuki M. Okamura N. Kawachi Y. Tashiro M. Arao H. Hoshishiba T. (2008). Discrete cortical regions associated with the musical beauty of major and minor chords. *Cogn. Affect. Behav. Neurosci.* 8 126–131. 10.3758/cabn.8.2.126 18589503

[B79] Thiede A. Glerean E. Kujala T. Parkkonen L. (2020). Atypical MEG inter-subject correlation during listening to continuous natural speech in dyslexia. *Neuroimage* 216:116799. 10.1016/j.neuroimage.2020.116799 32294536

[B80] Trost W. Ethofer T. Zentner M. Vuilleumier P. (2012). Mapping aesthetic musical emotions in the brain. *Cereb. Cortex* 22 2769–2783. 10.1093/cercor/bhr353 22178712 PMC3491764

[B81] Trost W. Frühholz S. Cochrane T. Cojan Y. Vuilleumier P. (2015). Temporal dynamics of musical emotions examined through intersubject synchrony of brain activity. *Soc. Cogn. Affect. Neurosci.* 10 1705–1721. 10.1093/scan/nsv060 25994970 PMC4666110

[B82] Tu P. C. Su T. P. Lin W. C. Chang W. C. Bai Y. M. Li C. T. (2019). Reduced synchronized brain activity in schizophrenia during viewing of comedy movies. *Sci. Rep.* 9:12738. 10.1038/s41598-019-48957-w 31484998 PMC6726596

[B83] Wilson S. M. Molnar-Szakacs I. Iacoboni M. (2008). Beyond superior temporal cortex: Intersubject correlations in narrative speech comprehension. *Cereb. Cortex* 18 230–242. 10.1093/cercor/bhm049 17504783

