# Supplementary material for: Inter-subject correlations of EEG reflect subjective arousal and acoustic features of music
Source: Front Hum Neurosci. 2023 Aug 21;17:1225377. doi: 10.3389/fnhum.2023.1225377 (PMC10475548; doi:10.3389/fnhum.2023.1225377)
Supplement: Supplementary file 1 [file Data_Sheet_1.docx]

Supplementary Material

**Inter-subject correlations of EEG reflect subjective arousal and acoustic features of music**

Fuyu Ueno and Sotaro Shimada^*^

*** Correspondence:** Sotaro Shimada; sshimada@meiji.ac.jp

# Supplementary Tables

**Supplementary Table 1.** Music lists.

| Clip | Ranking | Title | Year | Artist | Category |
| --- | --- | --- | --- | --- | --- |
| 1 | 1 | Koi | 2016 | Gen Hoshino | J-pop, Dance pop |
| 2 | 5 | Futarisezon | 2016 | Keyakizaka46 | J-pop |
| 3 | 10 | Silent majority | 2016 | Keyakizaka46 | J-pop |
| 4 | 15 | Negaigoto no motigusare | 2017 | AKB48 | J-pop |
| 5 | 20 | RAIN | 2017 | SEKAI NO OWARI | J-pop, Post-rock |
| 6 | 25 | Happy end | 2016 | back number | J-pop |
| 7 | 30 | EXCITE | 2017 | Daichi Miura | J-pop, R&B |
| 8 | 35 | Hikarinoatorie | 2017 | Mr.Children | J-pop, Rock |
| 9 | 40 | Doors -yuki no kiseki- | 2017 | Arashi | J-pop |
| 10 | 45 | Igai ni mango | 2017 | SKE48 | J-pop |
| 11 | 50 | HANABI | 2008 | Mr.Children | J-pop, Rock |
| 12 | 55 | CQCQ | 2017 | Kamisama boku ha kiduitesimatta | J-pop, Rock |
| 13 | 60 | KNOCK KNOCK | 2017 | TWICE | K-pop |
| 14 | 65 | Otona no okite | 2017 | Doughnuts Hole | J-pop |
| 15 | 70 | CHARM | 2017 | WANIMA | J-pop, Rock |
| 16 | 75 | Just You and I | 2017 | NAMIE AMURO | J-pop |
| 17 | 80 | Kisu ha matusikanainodeshouka? | 2017 | HKT48 | J-pop |
| 18 | 85 | Himawari no yakusoku | 2017 | Motohiro Hata | J-pop |
| 19 | 90 | Sora | 2017 | GENERATIONS from EXILE TRIBE | J-pop |
| 20 | 95 | BANG BANG BANG | 2016 | BIGBANG | K-pop, Dance pop, Trap, R&B, Seoul |
| 21 | 100 | Shake It Off | 2014 | Taylor Swift | Pop |

**Supplementary Table 2.** Music lists in each cluster: (A) Cluster 1, (B) Cluster 2, (C) Cluster 3.

| Clip | Ranking | Title | Year | Artist | Category |
| --- | --- | --- | --- | --- | --- |
| 1 | 1 | Koi | 2016 | Gen Hoshino | J-pop, Dance pop |
| 2 | 5 | Futarisezon | 2016 | Keyakizaka46 | J-pop |
| 9 | 40 | Doors -yuki no kiseki- | 2017 | Arashi | J-pop |
| 10 | 45 | Igai ni mango | 2017 | SKE48 | J-pop |
| 13 | 60 | KNOCK KNOCK | 2017 | TWICE | K-pop |
| 17 | 80 | Kisu ha matusikanainodeshouka? | 2017 | HKT48 | J-pop |
| 18 | 85 | Himawari no yakusoku | 2017 | Motohiro Hata | J-pop |

1. **Music lists in cluster 1.**
2. **Music lists in cluster 2.**

| Clip | Ranking | Title | Year | Artist | Category |
| --- | --- | --- | --- | --- | --- |
| 5 | 20 | RAIN | 2017 | SEKAI NO OWARI | J-pop, Post-rock |
| 6 | 25 | Happy end | 2016 | back number | J-pop |
| 8 | 35 | Hikarinoatorie | 2017 | Mr.Children | J-pop, Rock |
| 11 | 50 | HANABI | 2008 | Mr.Children | J-pop, Rock |
| 14 | 65 | Otona no okite | 2017 | Doughnuts Hole | J-pop |
| 16 | 75 | Just You and I | 2017 | NAMIE AMURO | J-pop |
| 21 | 100 | Shake It Off | 2014 | Taylor Swift | Pop |

1. **Music lists in cluster 3.**

| Clip | Ranking | Title | Year | Artist | Category |
| --- | --- | --- | --- | --- | --- |
| 3 | 10 | Silent majority | 2016 | Keyakizaka46 | J-pop |
| 4 | 15 | Negaigoto no motigusare | 2017 | AKB48 | J-pop |
| 7 | 30 | EXCITE | 2017 | Daichi Miura | J-pop, R&B |
| 12 | 55 | CQCQ | 2017 | Kamisama boku ha kiduitesimatta | J-pop, Rock |
| 15 | 70 | CHARM | 2017 | WANIMA | J-pop, Rock |
| 19 | 90 | Sora | 2017 | GENERATIONS from EXILE TRIBE | J-pop |
| 20 | 95 | BANG BANG BANG | 2016 | BIGBANG | K-pop, Dance pop, Trap, R&B, Seoul |

#
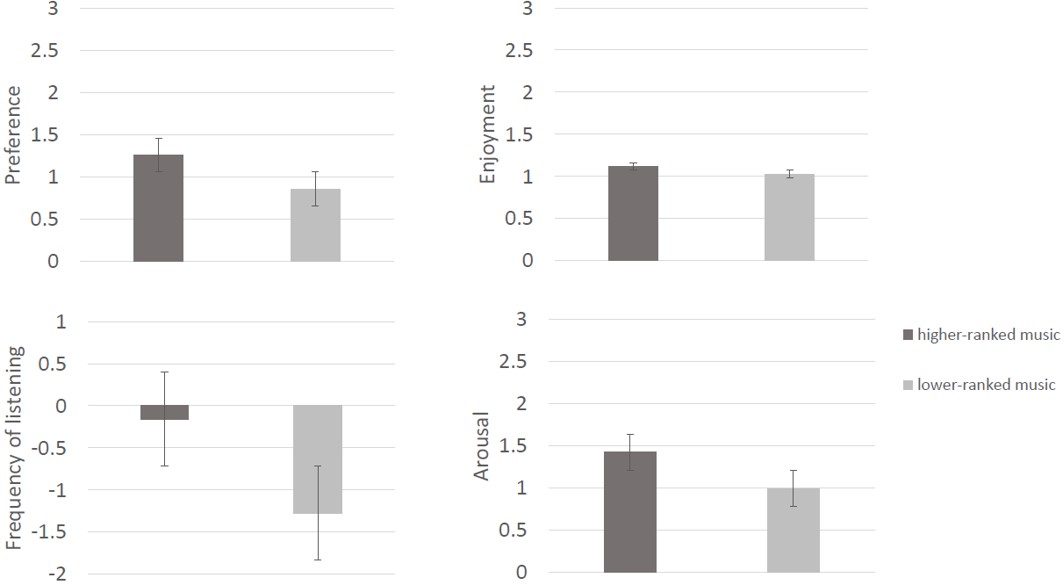
Supplementary Figures

**Supplementary Figure 1.** Subjects’ evaluation of higher-ranked and lower-ranked music on the chart.


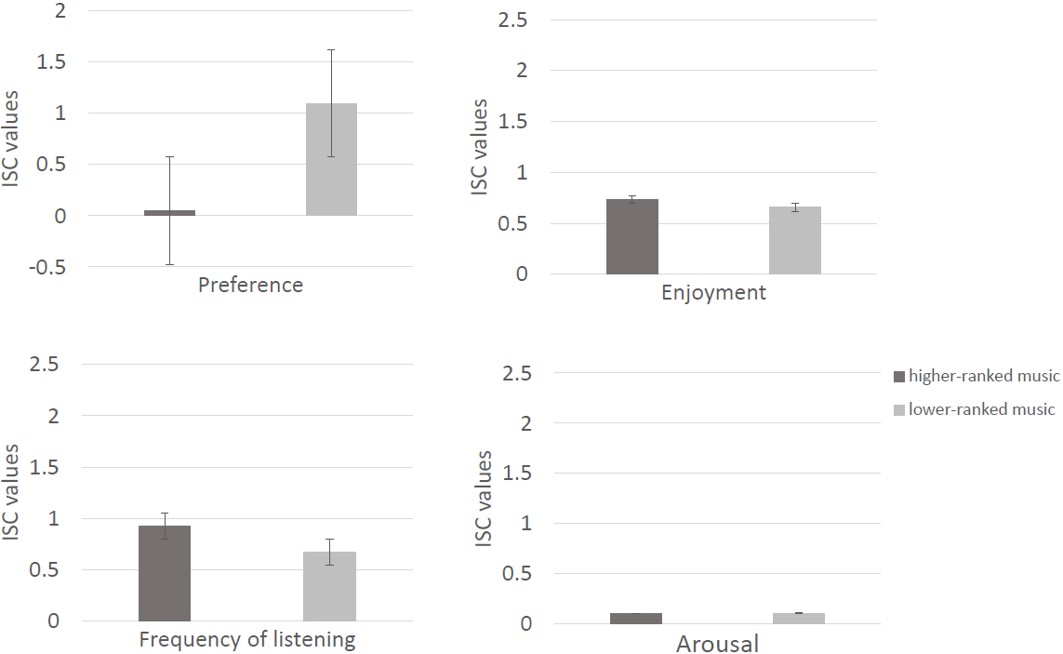


**Supplementary Figure 2.** Inter-subject correlation (ISC) values for higher-ranked and lower-ranked music according to subjects’ evaluation (preference, enjoyment, frequency of listening, and arousal).
